# The Influence of Hop Prenylated Chalcones on Mitochondrial Membrane Potential Depolarization and a Response to Oxidative Stress in MCC13 Merkel Cells

**DOI:** 10.3390/ph19050687

**Published:** 2026-04-27

**Authors:** Marcelina Chmiel, Aleksandra Włoch, Daniel Broda, Agata Bajek-Bil, Monika Stompor-Gorący

**Affiliations:** 1 Department of Organic Chemistry, Faculty of Medicine, University of Rzeszów, Rejtana 16C, 35-959 Rzeszów, Poland; monika.stompor@gmail.com; 2 Department of Physics and Biophysics, Wrocław University of Environmental and Life Sciences, Norwida 25, 50-375 Wrocław, Poland; aleksandra.wloch@upwr.edu.pl; 3 Faculty of Biotechnology, Collegium Medicum, University of Rzeszow, Pigonia 1, 35-310 Rzeszow, Poland; dbroda@ur.edu.pl; 4 Faculty of Chemistry, Rzeszów University of Technology, 35-959 Rzeszów, Poland; abajek@prz.edu.pl; 5 Department of Pathophysiology, Faculty of Medicine, University of Rzeszów, Rejtana 16C, 35-959 Rzeszów, Poland

**Keywords:** prenylated chalcones, xanthohumol, oxidative stress, mitochondrial membrane potential, Merkel cell carcinoma

## Abstract

**Background**: Prenylated chalcones are recognized for their beneficial nutritional properties and have attracted increasing interest due to their anticancer activities, which involve various mechanisms and pathways. In the current study, we investigated the influence of prenylated chalcone xanthohumol (XH) and its two minor derivatives xanthohumol C (XHC) and 1″,2″-dihydroxantohumol C (DHXHC) on the formation of reactive oxygen species (ROS), causing oxidative stress. Concomitantly, we studied the effect of mitochondrial transmembrane potential changes on human skin cancer, namely Merkel cell carcinoma (MCC13). **Methods**: The cancer cells were treated with the mentioned compounds for 24 and 48 h at various concentrations. **Results**: Our findings showed that ROS generation was dose-dependent at 24 h for xanthohumol, whereas for xanthohumol C and 1″2″-dihydroxanthohumol C, a significant increase in ROS occurred only at the highest concentration (100 μM) after 48 h. Mitochondrial membrane potential was significantly diminished by all the compounds. **Conclusions**: Taken together, our results indicate that the aforementioned chalcones exhibit cytotoxic activity against the MCC13 cell line and may be promising candidates for further investigation as anticancer agents.

## 1. Introduction

Natural compounds have gained a lot of interest in recent years for the treatment of multiple disorders, due to their multidirectional action and low toxicity [1,2]. Beneficial health properties have been reported for both natural flavones and modified flavanones, and also for their biosynthetic precursors—chalcones [3,4]. Various studies have confirmed their anticancer properties, owing to their cytotoxicity and proapoptotic activity [5,6]. Despite their low bioavailability and limited stability, they may be used in combination with other drugs to improve the effectiveness of pharmacotherapy [7].

Xanthohumol (3′-[3,3-dimethyl allyl]-2′,4′,4-trihydroxy-6′-methoxychalcone) is one of the best recognized chalcones isolated from the hop inflorescences of *Humulus lupulus* L., with the content ranging from 0.1 to 1%, depending on the variety [8,9]. It displays an array of biological properties such as antimicrobial [10,11], antioxidative [12], anti-inflammatory [13], and anti-diabetic properties [14], among many others [15,16,17].

Additionally, xanthohumol, as a chemopreventive agent, has been effective in multiple cancer types, such as breast [18] and lung cancers [19], glioblastoma [20], lymphoma, leukemia [21], colon [22], and gastric cancers [23]. Apart from xanthohumol itself, its various analogues have already been identified [24,25,26,27]; however, due to their limited availability, little is known about the pharmacological activity of these related compounds. Nevertheless, several syntheses that convert xanthohumol into its derivatives have been described in the literature [28,29,30].

Conversion of xanthohumol into xanthohumol C involves oxidative intramolecular cyclization of the prenyl side chain with the hydroxyl group at the 4′ position, resulting in the formation of the 2,2-dimethylchromene ring fused to the chalcone framework [31]. This cyclized form, named xanthohumol C, has shown multiple biological activities. As demonstrated by Kim et al. [32], XHC exhibited inhibitory effects on the monophenolase activity of mushroom tyrosinase, with an IC_50_ of 20.6 µM. Therefore, it might be a candidate for a skin-lightening agent. Other findings suggest that XHC strongly inhibits firefly luciferase in a dose-dependent manner [33]. Most studies evaluating xanthohumol C activity have been concentrated on its antibacterial and neuroprotective properties [34,35]. In 2018, Roehrer et al. [36] showed the moderate antimicrobial activity of XHC toward the Gram-positive bacteria *B. subtilis* and yeast *S. cerevisiae*, which depended on the pH of the media. Another study, conducted by Almuhayawi et al. [37], demonstrated that XHC has inhibitory potential against *E. xiangfangensis* by binding to all targeted proteins. Based on the latest research, it was confirmed that XHC is the most potent compound displaying neuroprotective activity against CoCl_2_-induced neuronal cell death [38]. Comparable results were observed in an in vivo study on a complex of xanthohumol C, supporting further investigation of this compound as a neuroprotective agent [39]. 1″,2″-Dihydroxanthohumol C has not gained as much popularity as the compounds mentioned above, yet it is confirmed that DHXHC is a very potent inducer of neuronal differentiation in MEF cultures and strongly activates the neuronal gene promoter DCX, suggesting that it acts at the early stage of neuronal differentiation [38].

Homeostasis is a fundamental state for proper cell function, optimal metabolism, growth, and survival. Factors that disrupt this balance can cause cellular dysfunction, including oxidative stress [40]. This arises from disequilibrium between the generation and accumulation of reactive oxygen species (ROS) in cells and tissues and the system’s ability to eliminate these products. Vasquez et al. [41] have proven the ability of xanthohumol to reduce ROS through the activation of Nrf2 (Nuclear factor erythroid 2-related factor 2), which is mainly characterized by the transfer of Nrf2 from the cytosol into the nucleus. Similar conclusions were drawn by the teams of Bai [42] and Yao [43], who additionally proved that the key determinant of the cytoprotective activity of xanthohumol is the α,β-unsaturated ketone structure.

Apart from oxidative stress, reprogramming of metabolic pathways in cancer cells is often accompanied by mitochondrial impairment [44]. Both phenomena are closely related; excessive production of ROS damages proteins, lipids, and mitochondrial DNA. This leads to a dysfunction of respiratory chain complexes and a decrease in the electrical potential difference across the inner mitochondrial membrane. Reduction in the membrane potential disrupts electron transportation, which is followed by an increased electron leakage and ROS production, creating a vicious circle [45]. This relation has been confirmed by Zhang et al. [46], who showed that xanthohumol directly interacts with the mitochondrial electron transfer chain complex I, triggering the production of ROS and inducing apoptosis. Consequently, exposure to xanthohumol causes a rapid decrease in mitochondrial transmembrane potential.

All these findings strongly suggest that XH and its analogues may be potential agents for the prevention and treatment of many diseases, especially those caused or exacerbated by oxidative stress, like cardiovascular and neurodegenerative diseases, diabetes, or cancers. Recently, we investigated xanthohumol and its selected analogues for antiproliferative effects against cancer cells, using the 72 h SRB assay, and the IC_50_ doses were determined [20]. The results of the research on the interactions of hop xanthohumols with lipid membranes and their toxicity to erythrocytes will be reported separately.

In the present study, we evaluated the effect of xanthohumol and its two derivatives (xanthohumol C and 1″,2″-dihydroxanthohumol C) on the rare and aggressive skin cancer that develops from neuroendocrine cells in the skin, most often on sun-exposed areas, which is Merkel cell carcinoma. We examined how these compounds modulate the mitochondrial membrane in cells and their ability to produce reactive oxygen species at two time points (24 and 48 h). This study is limited to in vitro exploration, while in vivo and clinical relevance have not yet been established. To the best of our knowledge, this is the first scientific report showing the effect of these derivatives on cell condition in MCC13 Merkel carcinoma.

## 2. Results

### 2.1. Mitochondrial Membrane Effect of Xanthohumol, Xanthohumol C, and 1″,2″-Dihydroxanthohumol C on MCC13 Cells Assessed by Mitopotential Assay

**Figure 1:**
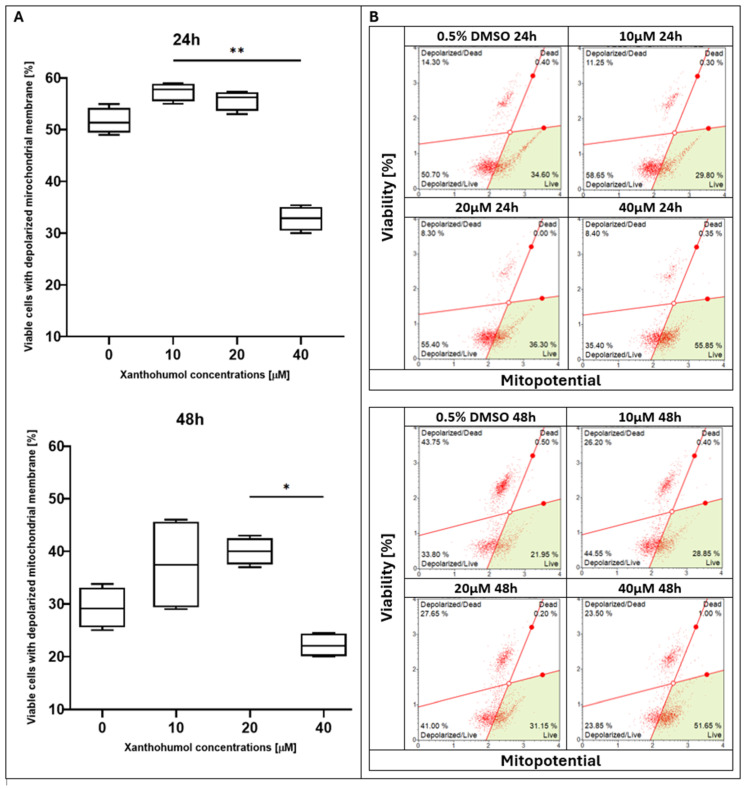
(**A**) Evaluation of percentage of depolarized/live cells in MCC13 cell line after treatment with xanthohumol for 24 and 48 h. All values are presented as medians using Friedman ANOVA *p* = 0.0001 and post hoc tests * *p* < 0.05 and ** *p* < 0.01. (**B**) Mitochondrial depolarization of MCC13 cells after treatment with xanthohumol for 24 and 48 h. Cells were analyzed using flow cytometry. Green area: live cells. Red solid lines separate four populations: live cells (**bottom right**), depolarized/live cells (**bottom left**), depolarized/dead cells (**top left**), and dead cells (**top right**).

**Figure 2:**
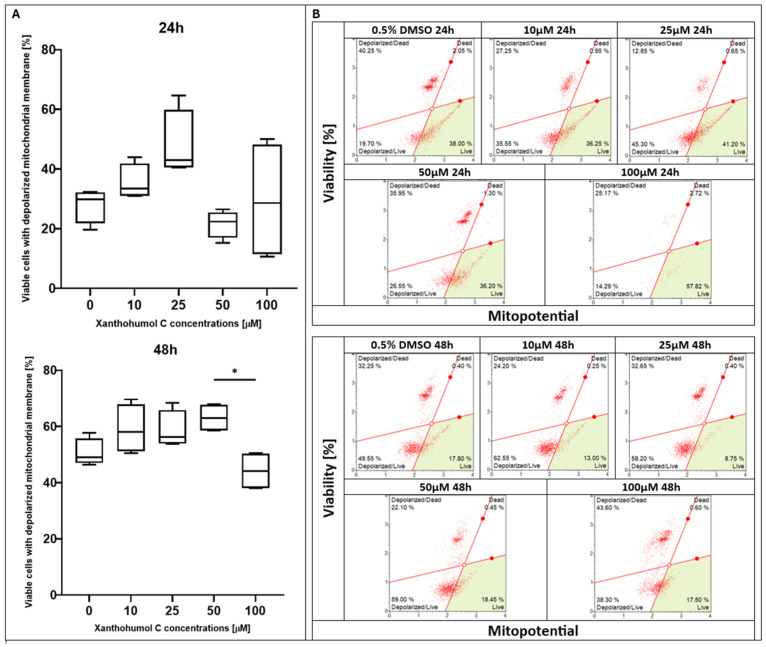
(**A**) Evaluation of percentage of depolarized/live cells in MCC13 cell line after treatment with xanthohumol C for 24 and 48 h. All values are presented as medians using Friedman ANOVA *p* = 0.0017 and post hoc tests * *p* < 0.05. (**B**) Mitochondrial depolarization of MCC13 cells after xanthohumol C treatment for 24 and 48 h. Cells were analyzed using flow cytometry. Green area: live cells. Red solid lines separate four populations: live cells (**bottom right**), depolarized/live cells (**bottom left**), depolarized/dead cells (**top left**), and dead cells (**top right**).

**Figure 3:**
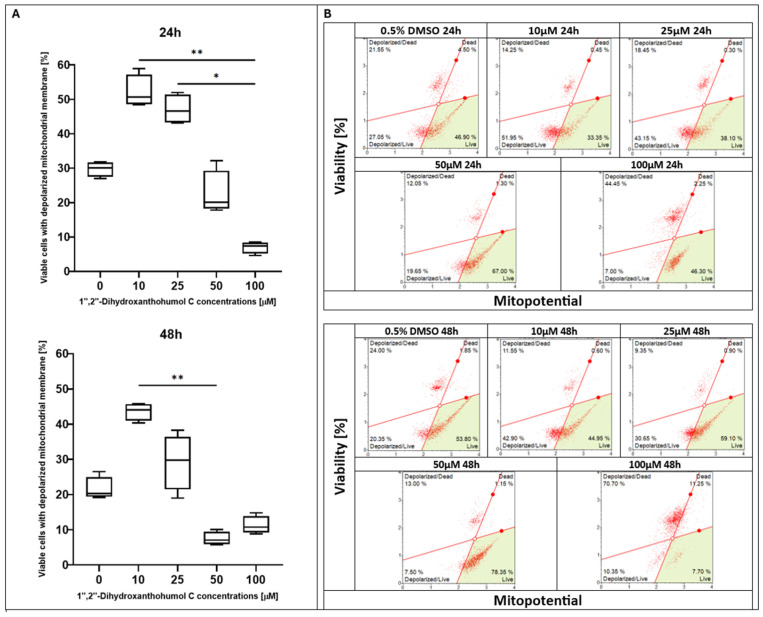
(**A**) Evaluation of percentage of depolarized/live cells in MCC13 cell line after treatment with 1″,2″-dihydroxanthohumol C for 24 and 48 h. All values are presented as medians using Friedman ANOVA *p* = 0.0001 and post hoc tests * *p* < 0.05 and ** *p* < 0.01. (**B**) Mitochondrial depolarization of MCC13 cells after 1″,2″-dihydroxanthohumol C treatment for 24 and 48 h, respectively. Cells were analyzed using flow cytometry. Green area: live cells. Red solid lines separate four populations: live cells (**bottom right**), depolarized/live cells (**bottom left**), depolarized/dead cells (**top left**), and dead cells (**top right**).

After 24 h of treatment with xanthohumol (XH), the highest percentage of viable cells with a depolarized membrane was observed at the dose of 10 µM; however, the control (0.5% DMSO) and 20 µM treatments showed similar levels. Nevertheless, a significant reduction in the percentage of live cells was noted in the culture supplemented with 40 μM of xanthohumol. Incubation for 48 h resulted in a decline in the living cell number at each concentration point compared to earlier the timepoint, whereas a statistically significant effect was noticed in the sample with the maximum XH concentration (Figure 1). Regarding xanthohumol C (XHC), no significant changes in the percentage of living cells were observed after 24 h of treatment; however, the fraction of depolarized/live cells showed a peak with the 25 µM concentration of XHC, and the lowest fraction was observed at the dose of 100 µM. A statistically significant effect was observed after 48 h of incubation with this compound. Three of the lowest doses of xanthohumol C were almost at the same level, while the lowest percentage of live cells was observed at 100 µM (Figure 2). In terms of 1″,2″-dihydroxanthohumol C (DHXHC), dose dependence was observed. After the 24 h treatment with this compound, the number of viable cells with depolarized membranes decreased proportionally to the increase in concentration of 1″,2″-dihydroxanthohumol C in the sample (from 10 to 100 µM). A notable difference was observed after 48 h of incubation. A dose-dependent decrease in cell viability was observed, and for each concentration, the viability was lower after 48 h of treatment compared to 24 h. A significant drop was observed at the 50 µM dose (Figure 3).

### 2.2. Oxidative Stress Effect of Xanthohumol, Xanthohumol C, and 1″,2″-Dihydroxanthohumol C on MCC13 Cells Assessed by Oxidative Stress Kit

**Figure 4:**
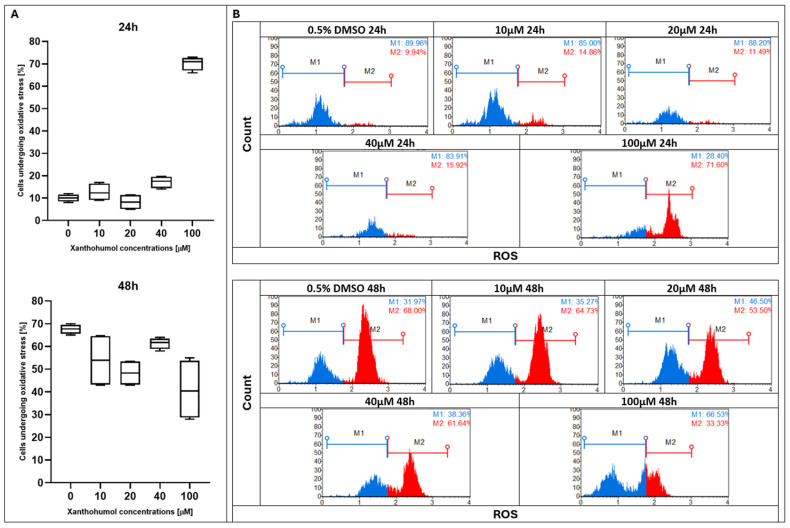
(**A**) Evaluation of percentage of ROS-positive MCC13 cells after treatment with xanthohumol at various concentrations. Statistical significance is indicated using Friedman ANOVA, *p* = 0.0005. (**B**) ROS profiles of MCC13 cell line after treatment with xanthohumol for 24 and 48 h. Histogram presents two cell populations: ROS(−) (blue line) and ROS(+) (red line) cells. Cells were analyzed using flow cytometry.

**Figure 5:**
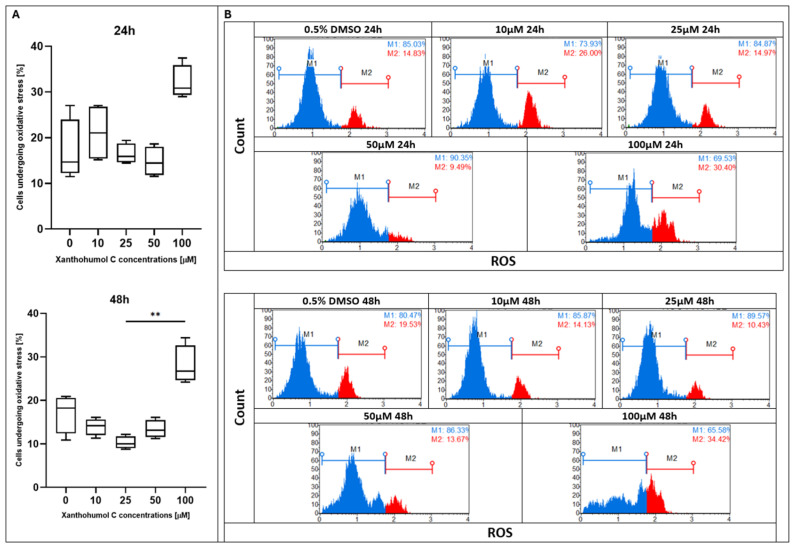
(**A**) Evaluation of percentage of ROS-positive MCC13 cells following treatment with xanthohumol C at various concentrations. Statistical significance is indicated using Friedman ANOVA *p* = 0.0008 and post hoc tests and ** *p* < 0.01. (**B**) ROS profiles of MCC13 cell line after treatment with xanthohumol C for 24 and 48 h. The histogram presents two cell populations: ROS(−) (blue line) and ROS(+) (red line) cells. Cells were analyzed using flow cytometry.

**Figure 6:**
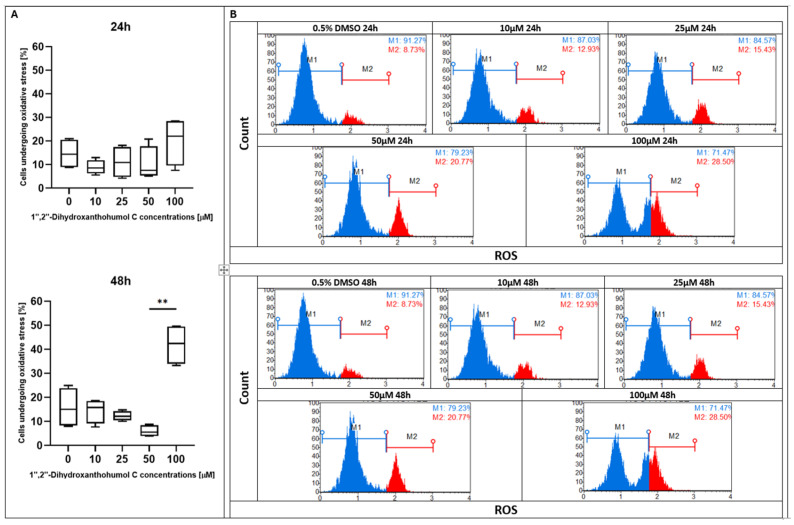
(**A**) Evaluation of the percentage of ROS-positive MCC13 cells following treatment with 1″,2″-dihydroxanthohumol C at various concentrations. Statistical significance is indicated using Friedman ANOVA *p* = 0.0051 and post hoc tests and ** *p* < 0.01. (**B**) ROS profiles of MCC13 cell line after treatment with 1″,2″-dihydroxanthohumol C for 24 and 48 h. The histogram presents two cell populations: ROS(−) (blue line) and ROS(+) (red line) cells. Cells were analyzed using flow cytometry.

During the first 24 h of treatment with xanthohumol at doses of 10, 20, and 40 µM, no increase in ROS production was observed compared to the control; however, the 100 µM dose significantly increased the percentage of cells undergoing oxidative stress. Surprisingly, after 48 h of incubation, the highest percentage of ROS generation was observed in the control sample and under 10, 20, and 40 µM of XH treatment, whereas 100 µM notably reduced the production of ROS (Figure 4). Similarly to xanthohumol, its derivative xanthohumol C had a stable level of ROS formation for 24 h of treatment, and only the dose of 100 µM slightly enhanced ROS production. After 48 h of supplementation, a significant difference was noted between the doses of 25 and 100 µM, whereas other doses did not change the generation of ROS (Figure 5). Under the treatment with 1″,2″-dihydroxanthohumol C, no effect of ROS overproduction was observed for the first 24 h. A meaningful change was noticed after 48 h of incubation, where a high growth in ROS generation appeared at 100 µM (Figure 6).

### 2.3. Examination of Antioxidant Capacity by ABTS•+ Scavenging Assay

In the ABTS•+ radical cation scavenging assay system, each compound was accurately weighed, and the concentrations were adjusted to achieve an absorbance of 0.70 at 734 nm. To determine the % of inhibition, a Trolox calibration curve was prepared with the equation Y = 0.1107X − 0.5565 and a highly positive linear correlation (R^2^ = 0.998). For this reason, the curve was applied to accurately evaluate the antioxidant capacity of the examined samples. In the ABTS assay, the compounds DHXHC and XHC presented the highest value of antioxidant capacity, with 2333.1 and 2318.0 µM Trolox/g D.M., respectively. A slightly lower Trolox value was observed for XH (1881.2 µM Trolox/g D.M) (Table 1).

## 3. Discussion

Increasing cancer morbidity is a current social issue. It is especially difficult to find effective therapies for new diseases with unknown etiology. Thus, a detailed analysis of the molecular basis of a disease is helpful for establishing therapeutic targets.

Merkel cell carcinoma (MCC) is a rare but aggressive skin cancer with frequent local recurrences and regional lymph node metastasis [47]. These cancer cells were originally discovered in 1873, whereas the disease was described for the first time by Toker in 1972 [48]. Merkel cells are found in the epidermis, where they act as skin sensory receptors. Merkel cell carcinoma may develop through two different oncogenic pathways. The first one is induced by the Merkel polyomavirus MCPyV (80% of cases), and the second is associated with excessive UV exposure leading to UV light-induced DNA damage and mutations (20% of cases). The disease often coexists with other malignancies, and importantly, in 50% of patients at the time of diagnosis, not only is regional lymph node metastasis present, but distant metastases are also present, with the liver, lungs, bone, and brain being involved. Currently, there is no approved therapeutic strategy for the efficient treatment of Merkel cell carcinoma [49]. In advanced stages of the disease, PD-1/PD-L1 inhibitors are used as first-line treatment drugs [50]. However, in 50% of cases, the results are not satisfactory. Therefore, there is a constant search for new, more effective therapies, such as adjuvant or neoadjuvant immunotherapy [51]. Because the growth and metastasis of cancers are strongly related to metabolic changes in cells, new therapeutic agents that can interrupt cellular metabolism in Merkel cells by disrupting mitochondrial function, changing redox potential, or inducing apoptosis may be an effective therapeutic strategy in the future.

In the literature, there are known examples of natural prenylchalcones’ anticancer effects on skin cancers [52]. For example, licochalcone D derived from *Glycyrrhiza inflata* inhibits human melanoma cell growth [53]. Hop’s xanthohumol has also been tested for clinical applications [54].

In this paper, we investigated the inhibitory effects of xanthohumol, xanthohumol C, and 1″,2″-dihydroxanthohumol C on the cell viability and oxidant activity of MCC13 Merkel cells. Structural alterations of the cell membrane represent a characteristic feature of apoptotic cell death. These changes might occur due to depolarization of the inner mitochondrial membrane potential and have become increasingly important in the study of apoptosis and drug toxicity. Disruption of this potential happens simultaneously with the opening of the transition pores, leading to a release of cytochrome c and triggering other apoptotic cascades, like oxidative stress [55].

This study provides evidence that xanthohumol significantly decreases the percentage of viable cells with depolarized mitochondrial membranes after 24 and 48 h of incubation. Cells treated with the lowest doses of XH (10 and 20 µM) had the highest percentage of viable cells at both timepoints. Although exposure to each of the tested XH concentrations affected the number of living cells, the highest dose (40 µM) caused a significant drop in cell viability. Taking into consideration ROS production, the cells supplemented with 100 µM of xanthohumol generated an overproduction of ROS compared to the control within the first 24 h, whereas the prolonged treatment reduced the percentage of the cells undergoing oxidative stress. The evaluation of the antioxidant activity of xanthohumol expressed as a Trolox equivalent indicates the significant antioxidant potential of this compound, although it is slightly lower compared to its derivatives: xanthohumol C and 1″,2″-dihydroxanthohumol C.

Research conducted by Pan et al. [56] proved that XH treatment (15 µM) leads to the activation of both intrinsic (mitochondrial) and extrinsic (death receptor) apoptotic pathways in human colon cancer cells after 24 and 48 h of incubation. Similarly, Yoo [57] and Zhang et al. [46] supported these findings by revealing that a rapid decrease in mitochondrial transmembrane potential was concomitant with overproduction of ROS. Furthermore, Grudzień et al. [21] found that 24 h treatment with xanthohumol led to a loss of membrane potential, together with an expansion of oxygen radicals. Further data demonstrate that upon treatment with xanthohumol, cancer cells showed a rapid but transient increase in superoxide generation. The ROS burst was correlated with mitochondrial dysfunction, primarily the loss of mitochondrial membrane potential through the release of cytochrome c and the activation of caspases. Thus, xanthohumol appears to use oxidative stress as a signalling trigger to engage the mitochondria-mediated apoptotic machinery in cancer cells [58]. However, in Żołnierczyk’s study [59], the other derivatives exhibited antioxidant activity comparable to or higher than that of xanthohumol, indicating that XH modifications may preserve or even enhance radical scavenging capacity in the ABTS assay. Nevertheless, our data are consistent with the literature reports on xanthohumol, which is described as an effective ABTS radical scavenger [60,61].

In the course of analyzing the XH derivatives, we found that xanthohumol C is a promising mitochondrial depolarizing agent in the MCC13 cell line, displaying and maintaining its activity after 48 h at a concentration of 100 µM. Longer incubation with the highest dose of XHC (100 µM) showed a higher percentage of dead cells with a depolarized mitochondrial membrane, while the lower doses were not that efficient. Loss of the mitochondrial inner transmembrane potential is frequently observed in the early stages of apoptosis. Additionally, we evaluated the effect of XHC treatment on ROS production in Merkel cells, which showed encouraging activity after 24 h. A significant difference was observed in the case of supplementation with 100 µM of XHC, where the extended exposure resulted in a stable proportion of the cells undergoing oxidative stress (about 30%). It is also noteworthy that xanthohumol C demonstrated antiproliferative and cytotoxic effects. In human stomach and hepatic carcinomas, XHC had marginal cytotoxic activity against these cell lines [26]; however, robust outcomes were provided by the Roehrer group [62] in breast cancer cells (MCF-7). They demonstrated that XHC had the best growth inhibition effect of all the tested compounds, causing cell death at a concentration of 5 μM. The proteomic study proved that XHC strongly modulates proteins involved in endoplasmic reticulum (ER) stress, which is a known trigger of apoptotic pathways under prolonged or unresolved stress.

Along with xanthohumol C, 1″,2″-dihydroxanthohumol C displays favourable biological activity in various cancer cell lines. In our study, we confirmed that DHXHC had a significant impact on the depolarization of the mitochondrial membrane in MCC13 cells. After incubation for 24 h, the lowest percentage of viable cells was noted at the highest compound concentration (100 µM) and remained steady after 48 h. Interestingly, treatment with the 50 µM dose showed comparable results to the control treatment after the first 24 h, yet longer supplementation significantly reduced the percentage of viable cells, being slightly stronger than the dose of 100 µM. Furthermore, while treatment with DHXHC did not influence the cells regarding ROS generation for the first 24 h, a substantial increase was observed after 48 h. The 100 µM dose contributed to a high increase in ROS formation from 20 up to 40% (after 24 and 48 h, respectively). The results covering antioxidant activity demonstrate the strong ability of both xanthohumol C and 1″,2″-dihydroxanthohumol C to neutralize ABTS•+ radicals. In the literature, there is a limited amount of information regarding the activity of 1″,2″-dihydroxanthohumol C. In the study by Vogel and Heilmann [29], the authors demonstrated the antioxidant activity of 1″,2″-dihydroxanthohumol C in the oxygen radical absorbance capacity assay (ORAC). The Trolox equivalent value for this compound is 1.7 µM, and it has a similar activity to that of xanthohumol C and xanthohumol, with IC_50_ values of 1.8 and 2.3 µM, respectively. Additionally, the authors measured DHXHC’s cytotoxicity against HeLa cells using the MTT cell proliferation assay for 72 h. The results showed the promising antiproliferative activity of DHXHC (IC_50_ 15.4 µM), comparable to xanthohumol C and xanthohumol (IC_50_ 12.5 and 9.4 µM, respectively). Similar conclusions concerning the antiproliferative effects of DHXHC were drawn by Popłoński et al. [31], who measured the activity of this compound in human cancer cells, PC-3, HT-29, and MCF-7. The measured IC_50_ values were 49.6, 16.7, and 15.9 µM, respectively.

While the previously reported biological activities of xanthohumol C (**2**) and 1″,2″-dihydroxanthohumol C (**3**) are of significant interest, no studies to date have directly investigated their effects on mitochondrial membrane integrity or reactive oxygen species (ROS) production in any model. Our study is the first to demonstrate this effect in the MCC13 Merkel cell carcinoma cell line.

## 4. Materials and Methods

### 4.1. Cell Culture and Reagents

MCC13 cell line was purchased from the European Collection of Authenticated Cell Cultures (ECACC, Salisbury, UK). Cells were cultured in RPMI medium (Cat. #31870025, Gibco, Waltham, MA, USA) with 25 mM of HEPES (Cat. #15630080, Gibco, Waltham, MA, USA), supplemented with HyClone 15% fetal bovine serum (GE Healthcare, Chicago, IL, USA) and 2 mM of L-glutamine (Sigma-Aldrich, Steinheim, Germany). To prevent bacterial contamination, 100 U/mL of penicillin (Polfa Tarchomin, Warsaw, Poland) and 0.1 mg/mL of streptomycin (Sigma Aldrich, Steinheim, Germany) were used. For the experiments, the cells were seeded in flat-bottom 24-well culture plates (VWR, Radnor, PA, USA) with four biological repeats at a density of 1 × 10^4^ cells/well. The cells were allowed to attach for 24 h before treatment. For the entirety of the experiment, the cells were cultured in a humid atmosphere at 37 °C and 5% CO_2_.

### 4.2. Treatment of Cells

Xanthohumol (XH) was isolated from spent hop pellets obtained from the New Chemical Syntheses Institute in Puławy, Poland. Specifically, the material used for xanthohumol isolation was the byproduct of the industrial extraction of hop (variety Magnat) with supercritical carbon dioxide. Gradient-grade purity methanol was purchased from Merck (Darmstadt, Germany). All the other reagents, unless otherwise specified, were purchased from Sigma-Aldrich (St. Louis, MO, USA) and were of analytical grade.

Xanthohumol C (XHC) and 1″2″-dihydroxanthohumol C (DHXHC) were obtained by cyclisation of xanthohumol. Modifying the procedure described by Vogel and Heilmann [29], from the reaction of XH with DDQ (2,3-dichloro-5,6-dicyano-1,4-benzoquinone) dissolved in anhydrous 1,4-dioxane at 95 °C, we obtained xanthohumol C in 72% yield. DHXHC was obtained according to the modified method described earlier [63]. XH was stirred with anhydrous aluminum chloride in methylene chloride for 5 h at room temperature to afford DHXHC in 87% yield. The spectral data of the derivatives are consistent with the literature for XH in DMSO-d_6_ [64] and for XHC and DHXHC in acetone-d_6_ [31], respectively (See Supplementary Materials).

**Figure 7:**
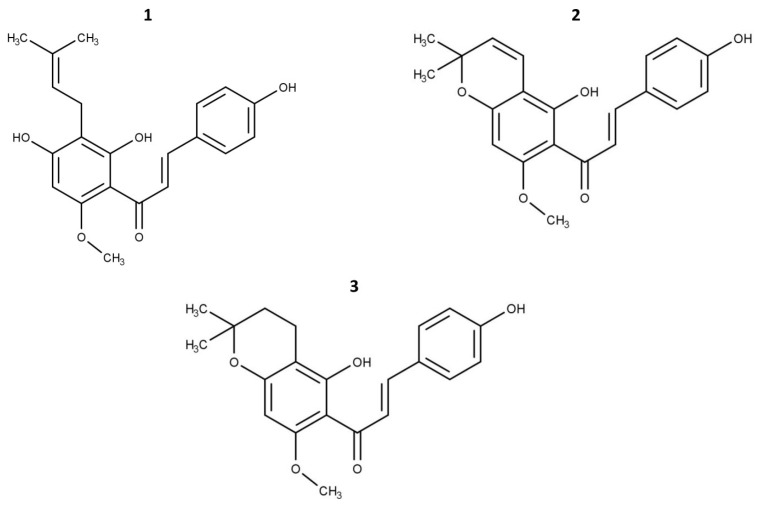
Chemical structures of tested compounds: (**1**) xanthohumol; (**2**) xanthohumol C; (**3**) 1″,2″-dihydroxanthohumol C. Chemical structures were drawn using MarvinSketch 25.5.0 (ChemAxon, Budapest, Hungary).

Xanthohumol (XH, C_21_H_22_O_5_, MW = 354.4), xanthohumol C (XHC, C_21_H_20_O_5_, MW = 352.4), and 1″,2″-dihydroxanthohumol C (DHXHC, C_21_H_22_O_5_, MW = 354.5) (Figure 7) were dissolved in dimethyl sulfoxide (DMSO) (VWR, Radnor, PA, USA) to produce 100 mM stock solutions. Microliter volumes of stock solutions were added to 1 mL of culture medium to obtain a range of concentrations of the tested compounds. The control solution contained 0.5% DMSO. For all compound concentrations, the final DMSO concentration was adjusted to 0.5% (*v*/*v*) by adding neat DMSO. The cells were maintained in the media for 24 and 48 h.

### 4.3. Determination of Changes in Mitochondrial Membrane Potential

To detect changes in mitochondrial membrane potential, the cells were subjected to the MitoPotential Assay (Cat.#MCH100110, Merck Millipore, Burlington, MA, USA) according to the manufacturer’s protocol. All measurements were conducted on a Muse Cell Analyzer (Merck Millipore, Burlington, MA, USA). MCC13 cells were treated with increasing concentrations of the xanthohumols and DMSO for 24 and 48 h. The cells were collected, washed, and incubated with Mitopotential Dye, a fluorescent and lipophilic dye used to measure the mitochondrial membrane changes in live cells, for 20 min at 37 °C in the MS-100 Thermo Shaker block (Eppendorf, Hamburg, Germany). Then, the cells were incubated with 7-AAD, an indicator of dead cells, for 5 min at room temperature.

### 4.4. Detection of Oxidative Stress

The quantity of the superoxide radicals in the cells was measured using the Muse Cell Analyzer (Cat.#MCH100111, Merck Millipore, Burlington, MA, USA) with the Oxidative Stress Kit (Merck Millipore, Burlington, MA, USA), according to the manufacturer’s protocol. MCC13 cells were treated with increasing concentrations of xanthohumols (**1**–**3**) and DMSO for 24 and 48 h. Then, the cells were harvested, washed, and mixed with Muse Oxidative Stress Reagent working solution by vortexing at medium speed for 5 s. Next, the samples were incubated for 30 min at 37 °C in the MS-100 Thermo Shaker block (Eppendorf, Hamburg, Germany). The incubation was performed protected from light.

### 4.5. Antioxidant Capacity

The antioxidant capacities of the examined compounds were evaluated using the ABTS assay [65]. The method was adapted for microplate assays with some modifications. To prepare ABTS (2,2′-azino-bis-3-ethylbenzthiazoline-6-sulphonic acid) stock solution, ABTS powder was dissolved in ultrapure water at a final concentration of 7 mM and mixed with potassium persulphate (4.9 mM) in a 1:1 ratio. The ABTS working solution was freshly prepared by diluting ABTS^+^ with methanol to achieve an absorbance of 0.70 at 734 nm. Then, 190 µL of ABTS+ working solution was mixed with 10 µL of the sample/standard/control solutions and incubated for 5 min in the dark. The absorbance was measured at 734 nm and compared to a Trolox equivalent calibration curve. The results were expressed as mM Trolox equivalents per g (mM Trolox/g of dry matter).

### 4.6. Statistical Analysis

For all assays, four technical replicates per condition were prepared, and the results of the analyses were presented as medians. The gating was set based on untreated samples to establish thresholds. The statistical analysis included ANOVA (Analysis of variance), Friedman, and post hoc tests. Calculations were performed using GraphPad Prism 10.4.1 (GraphPad Software, Inc., San Diego, CA, USA).

## 5. Conclusions

In this study, we explored the anticancer activity of xanthohumol and its two derivatives, xanthohumol C and 1″,2″-dihydroxanthohumol C, exploring their mitochondria-modulating properties and their effect on the generation of reactive oxygen species in MCC13 Merkel cell carcinoma after 48 h of incubation. All the tested compounds altered the mitochondrial membrane potential, showing promising anticancer properties. It is worth highlighting that among the tested compounds, 1″,2″-dihydroxanthohumol C displayed the highest effectiveness for ROS generation after 48 h of treatment, which demonstrates that cyclization of the prenyl group markedly enhances and stabilizes pro-oxidant activity compared to xanthohumol and xanthohumol C. Moreover, only xanthohumol exerted a different effect over time regarding ROS production. This suggests that xanthohumol might act as an ROS inducer over a short period and as an antioxidant when supplemented for longer. Taken together, these data indicate that prenylated hop chalcones could potentially be used as chemotherapeutic agents, and as either pro- or anti-oxidants depending on their optimal doses and time-dependent effects.

Our research contributes to a better understanding of the effects of xanthohumols on the cells of rare cancers, such as Merkel cell carcinoma. Also, it delivers information on how the modifications of the prenyl group affect the antitumor activity of hop xanthohumols against the MCC13 skin cancer cells. However, there is a need for further multi-aspect analysis of molecular mechanisms such as molecular validation of caspases, cytochrome c, or Bcl-2 family proteins. Examination of the antitumor activity of the tested compounds using other cell models, including head and neck cancers, is recommended. Additionally, an important avenue is testing the activity of hydroxychalcones without the prenyl group, for comparison. Then, the most promising selected chalcone structures could potentially be used to develop new drug formulations or may find clinical application as substances to enhance cancer treatment effectiveness.

## Figures and Tables

**Table 1:** Antioxidant capacity of the tested compounds measured by the ABTS assay.

Compounds	µM Trolox/g D.M.
	ABTS
Xanthohumol	1881.2
Xanthohumol C	2318.0
1″,2″-Dihydroxanthohumol C	2333.1

## Data Availability

The original contributions presented in this study are included in the article/Supplementary Materials. Further inquiries can be directed to the corresponding author.

## Supplementary Materials

The following supporting information can be downloaded at: https://www.mdpi.com/article/10.3390/ph19050687/s1, Figure S1: HR-ESI-MS mass spectra of xanthohumol (1), xanthohumol C (2) and 1′’,2′’-dihydroxanthohumol C (3); Figure S2: HPLC chromatograms and UV spectra of xanthohumol (1), xanthohumol C (2) and 1′’,2′’-dihydroxanthohumol C (3) with the percentage content of tested compounds; Figure S3: ^1^H-NMR spectrum of xanthohumol (1) in acetone-d_6_; Figure S4: ^13^C-NMR spectrum of xanthohumol (1) in acetone-d_6_; Figure S5: ^1^H-NMR spectrum of xanthohumol C (2) in acetone-d_6_; Figure S6: ^13^C-NMR spectrum of xanthohumol C (2) in acetone-d_6_; Figure S7: ^1^H-NMR spectrum of 1′’,2′’-dihydroxanthohumol C (3) in acetone-d_6_; Figure S8: ^13^C-NMR spectrum of 1′’,2′’-dihydroxanthohumol C (3) in acetone-d_6_.

## Abbreviations

The following abbreviations are used in this manuscript:

ANOVA	Analysis of variance
DHXHC	1″,2″-Dihydroxantohumol C
HeLa	Cervical carcinoma
HT-29	Colorectal adenocarcinoma cell line
MCC13	Merkel cell carcinoma
MCF-7	Breast cancer cells
PC-3	Prostate cancer cells
ROS	Reactive oxygen species
Nrf2	Nuclear factor erythroid 2-related factor 2
DDQ	2,3-dichloro-5,6-dicyano-1,4-benzoquinone
ABTS	2,2′-azino-bis-3-ethylbenzthiazoline-6-sulphonic acid
XH	Xanthohumol
XHC	Xanthohumol C
