# Supplementary material for: The Influence of Hop Prenylated Chalcones on Mitochondrial Membrane Potential Depolarization and a Response to Oxidative Stress in MCC13 Merkel Cells
Source: Pharmaceuticals (Basel). 2026 Apr 27;19(5):687. doi: 10.3390/ph19050687 (PMC13209938; doi:10.3390/ph19050687)
Supplement: Supplementary file 1 [file pharmaceuticals-19-00687-s001.zip › pharmaceuticals-4239396-supplementary.pdf]

Supplementary Materials: The Influence of Hop Prenylated Chalcones on Mitochondrial Membrane Potential Depolarization and a Response to Oxidative Stress in MCC13 Merkel cells

Marcelina Chmiel, Aleksandra Włoch, Daniel Broda, Agata Bajek-Bill and Monika Stompor-Gorący

Figure S1. HR-ESI-MS mass spectra of xanthohumol (**1**), xanthohumol C (**2**) and 1′′,2′′-dihydroxanthohumol C (**3**).

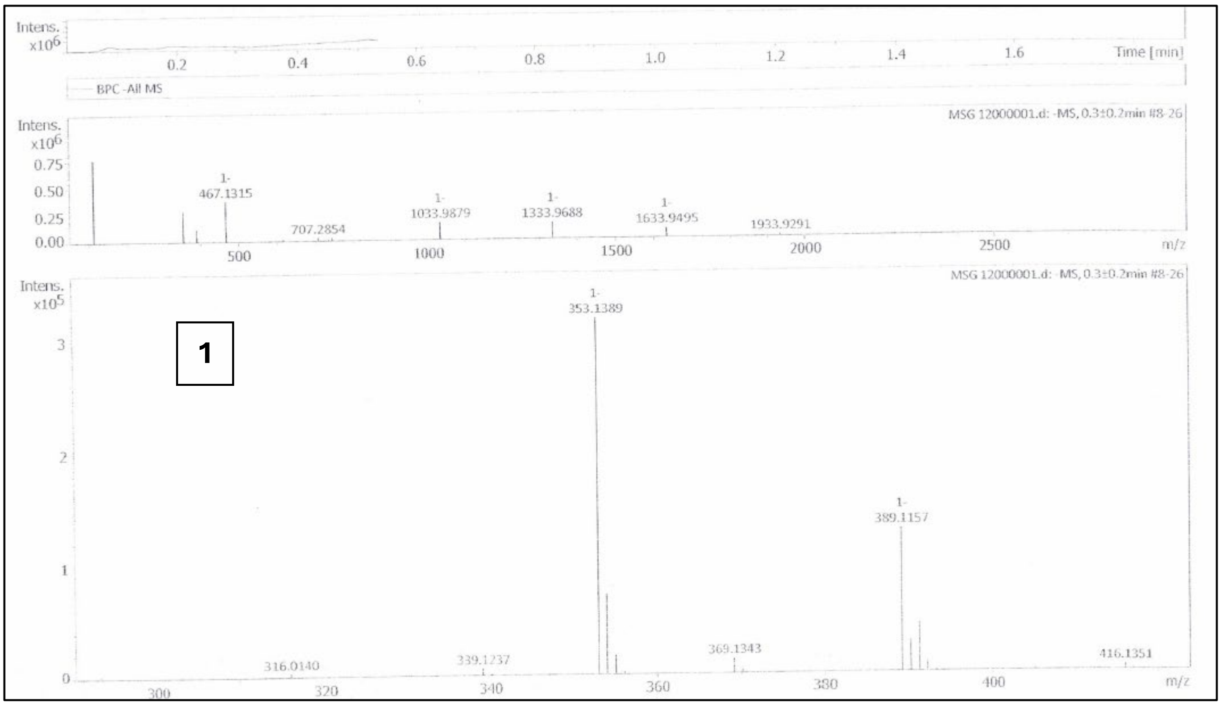

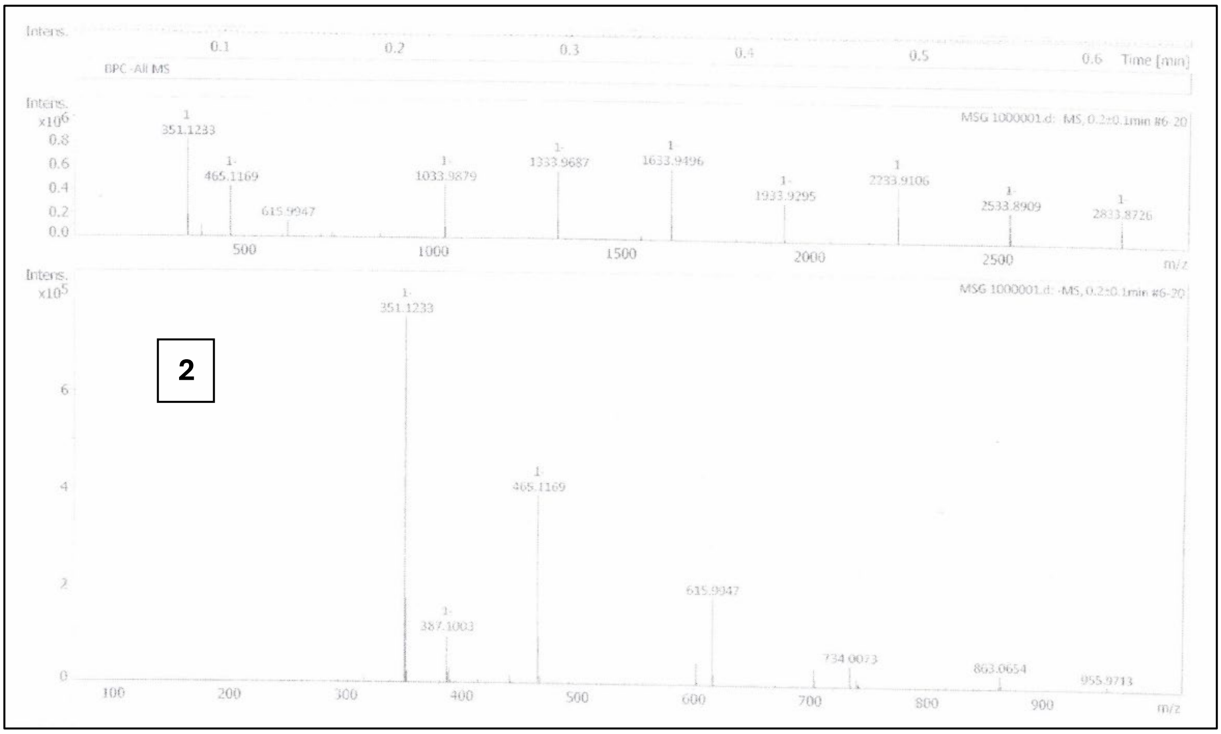

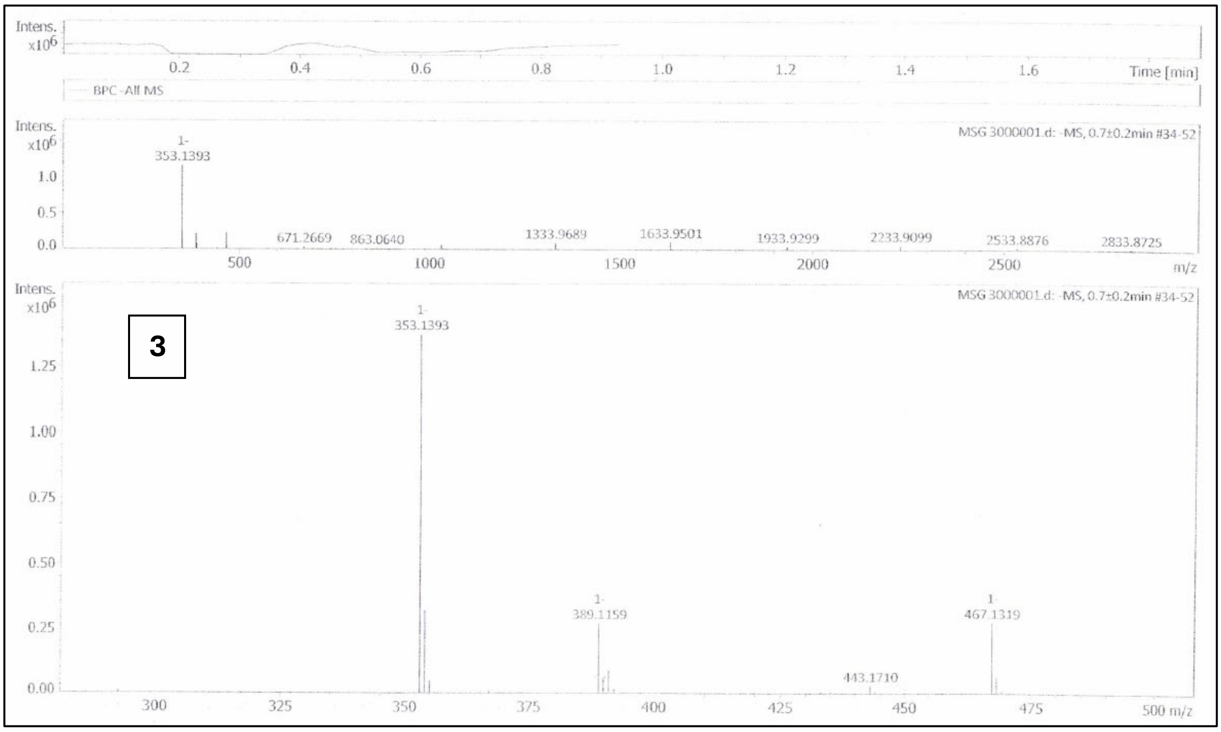

Figure S2. HPLC chromatograms and UV spectra of xanthohumol (**1**), xanthohumol C (**2**) and 1′′,2′′-dihydroxanthohumol C (**3**) with the percentage content of tested compounds.

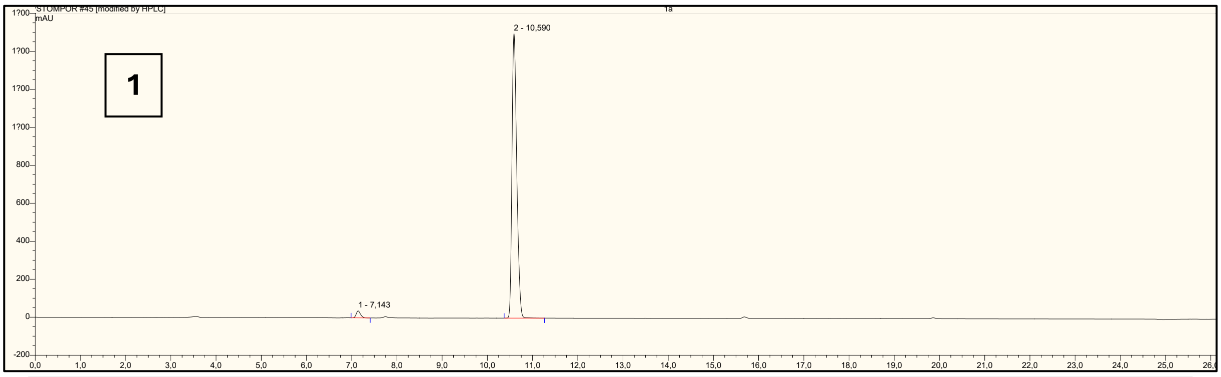

No.	Peakname	Ret.Time min	Area mAU*min	Amount	Type	Heigh mAU	Rel.Area %	Resolution
1	n.a	7,143	4,0340	n.a.	BMB	36,605	2,15	19,00
2	n.a	10,59	184,0052	n.a.	BMB	1496,441	97,85	n.a.
**Total:**			**188,0392**	**0,0000**		**1533,046**	**100,00**	

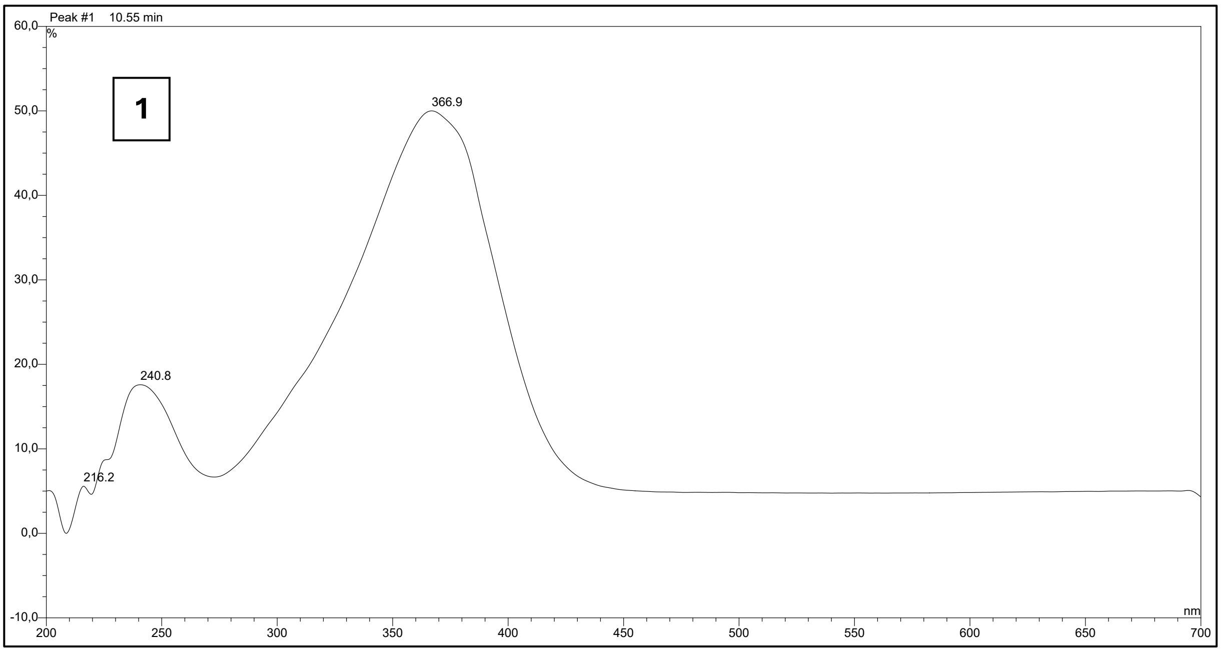

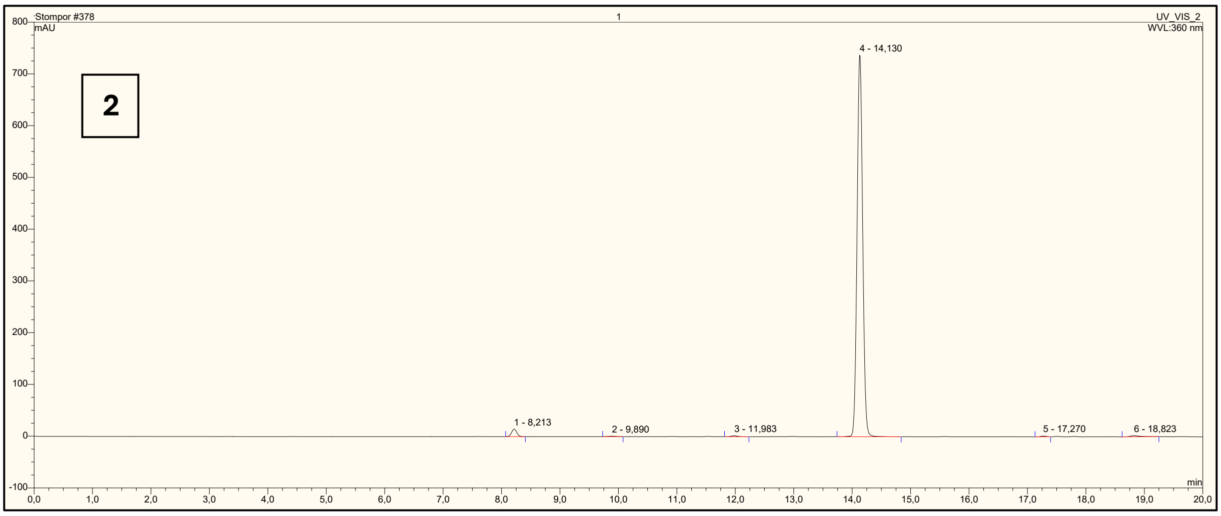

No.	Peakname	Ret.Time min	Area mAU*min	Amount	Type	Height mAU	Rel.Area %	Resolution
1	n.a.	8,213	1,4967	n.a.	BMB	14,444	1,78	9,36
2	n.a.	9,890	0,1192	n.a.	BMB	0,965	0,14	11,31
3	n.a.	11,983	0,2163	n.a.	BMB	1,901	0,26	12,24
4	n.a.	14,130	81,9172	n.a.	BMB	736,955	97,16	18,30
5	n.a.	17,270	0,1506	n.a.	BMB	1,441	0,18	7,16
6	n.a.	18,823	0,4118	n.a.	BMB	2,108	0,49	n.a.
Total:			84,3118	0,0000		757,814	100,00	

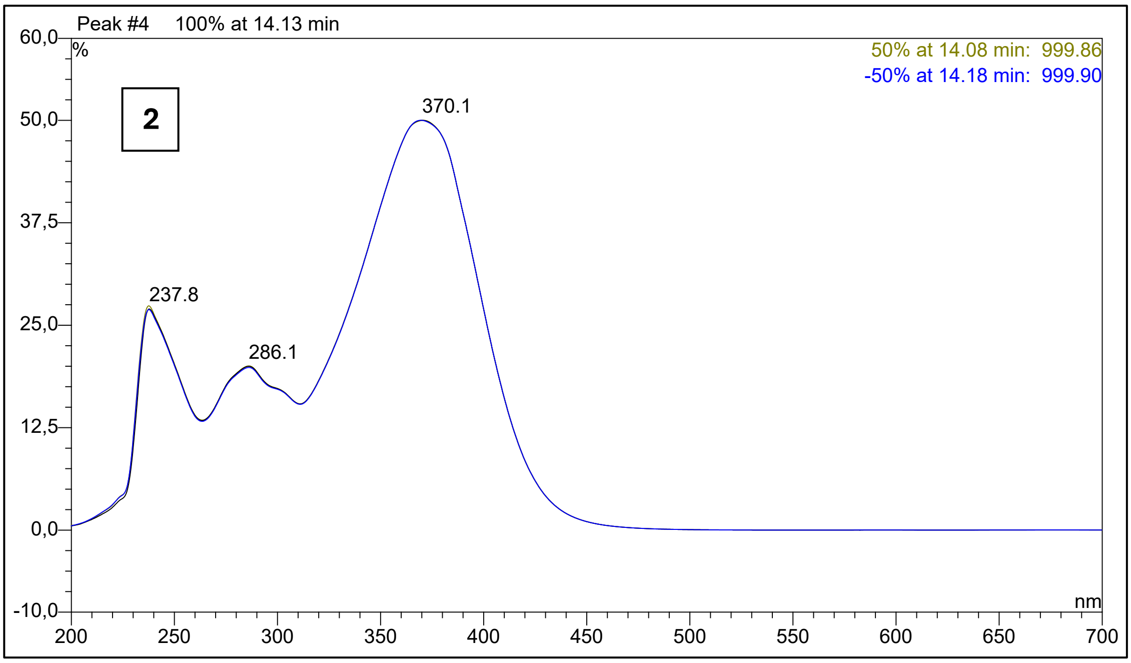

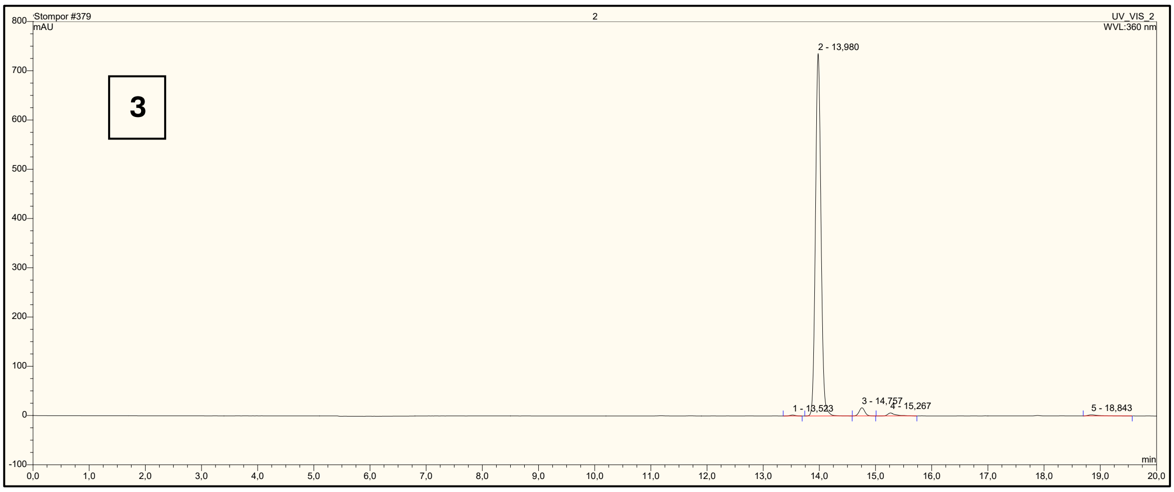

No.	Peakname	Ret.Time min	Area mAU*min	Amount	Type	Height mAU	Rel.Area %	Resolution
1	n.a.	13,523	0,2098	n.a.	BMB	1,868	0,24	2,58
2	n.a.	13,980	83,7800	n.a.	BMB	735,537	95,87	4,32
3	n.a.	14,757	1,9077	n.a.	BMB	16,482	2,18	2,66
4	n.a.	15,267	1,0038	n.a.	BMB	6,061	1,15	14,63
5	n.a.	18,843	0,4905	n.a.	BMB	2,138	0,56	n.a.
Total:			87,3918	0,0000		762,086	100,00	

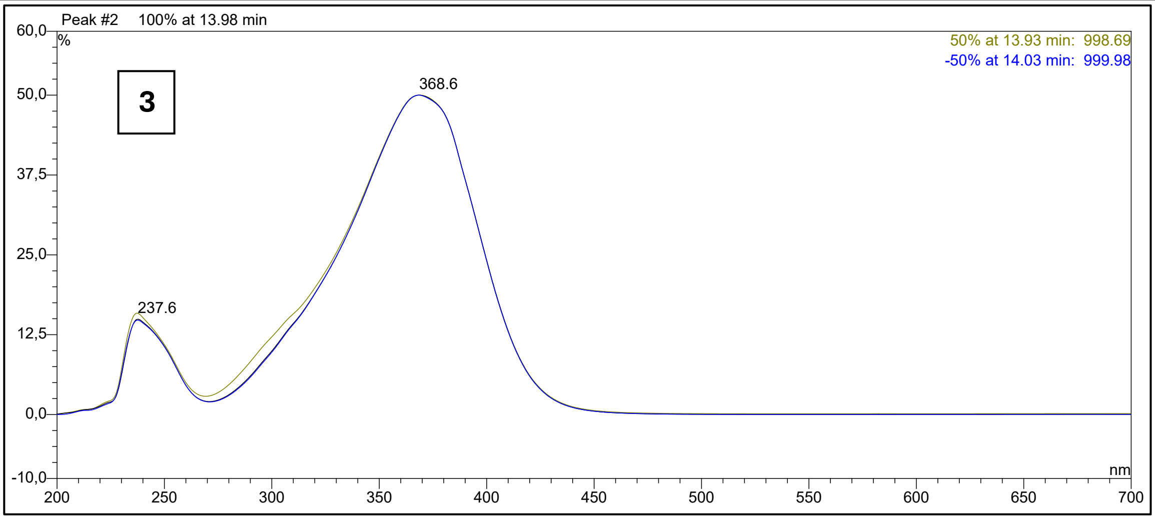

NMR spectra (^{1}H-NMR, ^{13}C-NMR, COSY, HSQC HMBC) of compounds 1-3 were recorded on a Bruker AVANCEII 500 NMR spectrometer (500 MHz for ^{1}H spectra and 125 MHz for ^{13}C spectra) in acetone-d_6 and/or DMSO-d_6,that served as an internal standard.

Figure S3. ^{1}H-NMR spectrum of xanthohumol (**1**) in acetone-d_6:

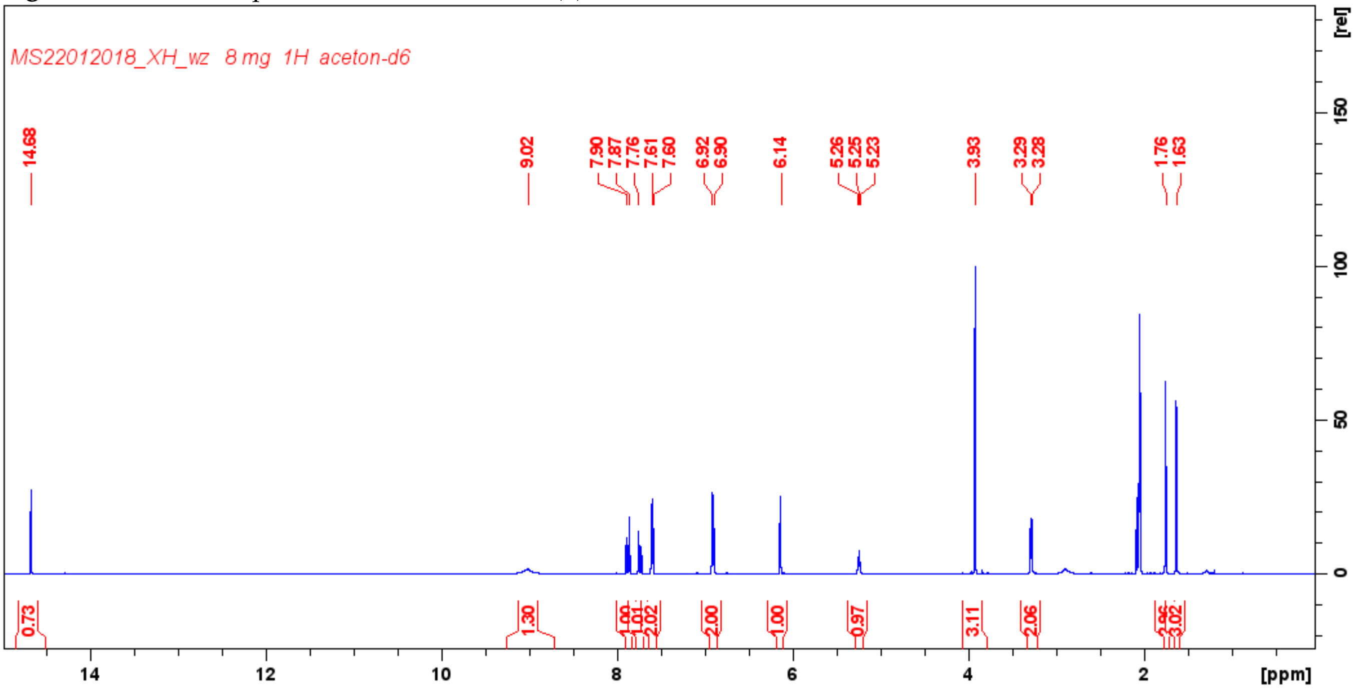

^{1}H-NMR spectrum in accordance with the literature data: https://www.chemicalbook.com/SpectrumEN_569-83-5_1HNMR.htm

Figure S4. ^{13}C-NMR spectrum of xanthohumol (1) in acetone-d_6:

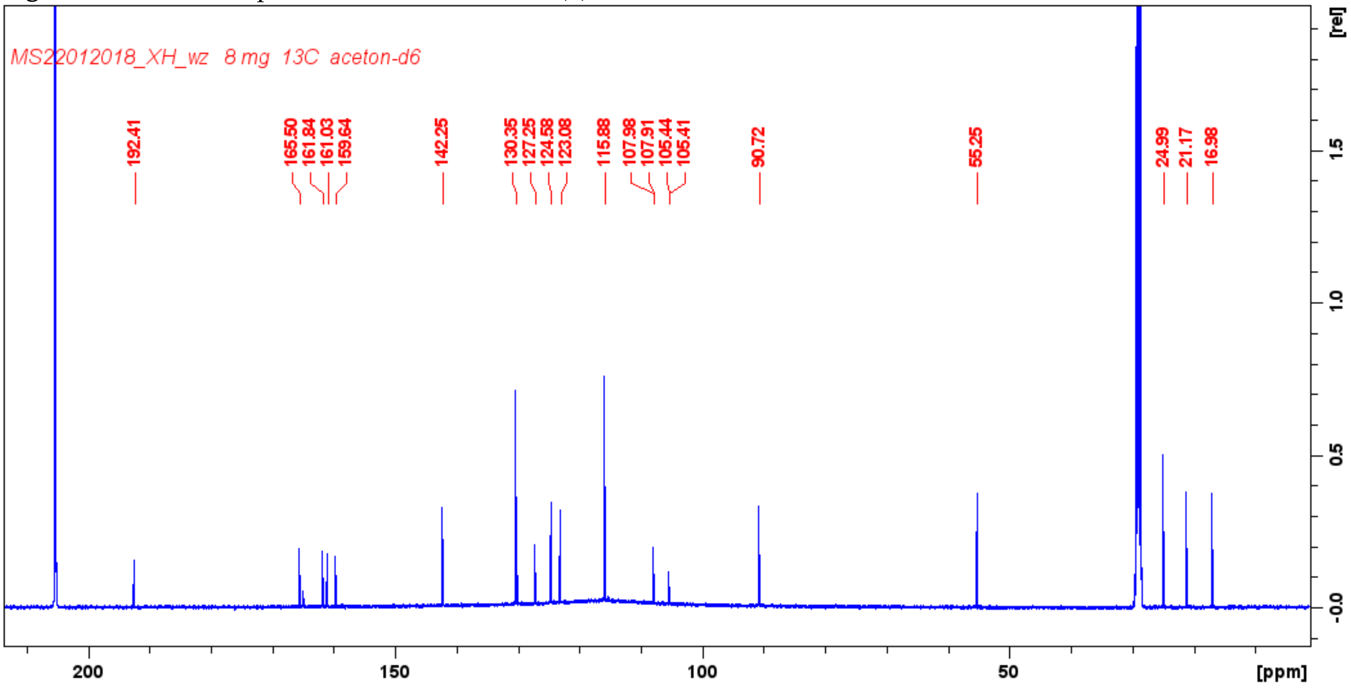

Figure S5. ^{1}H-NMR spectrum of xanthohumol C (**2**) in acetone-d_6:

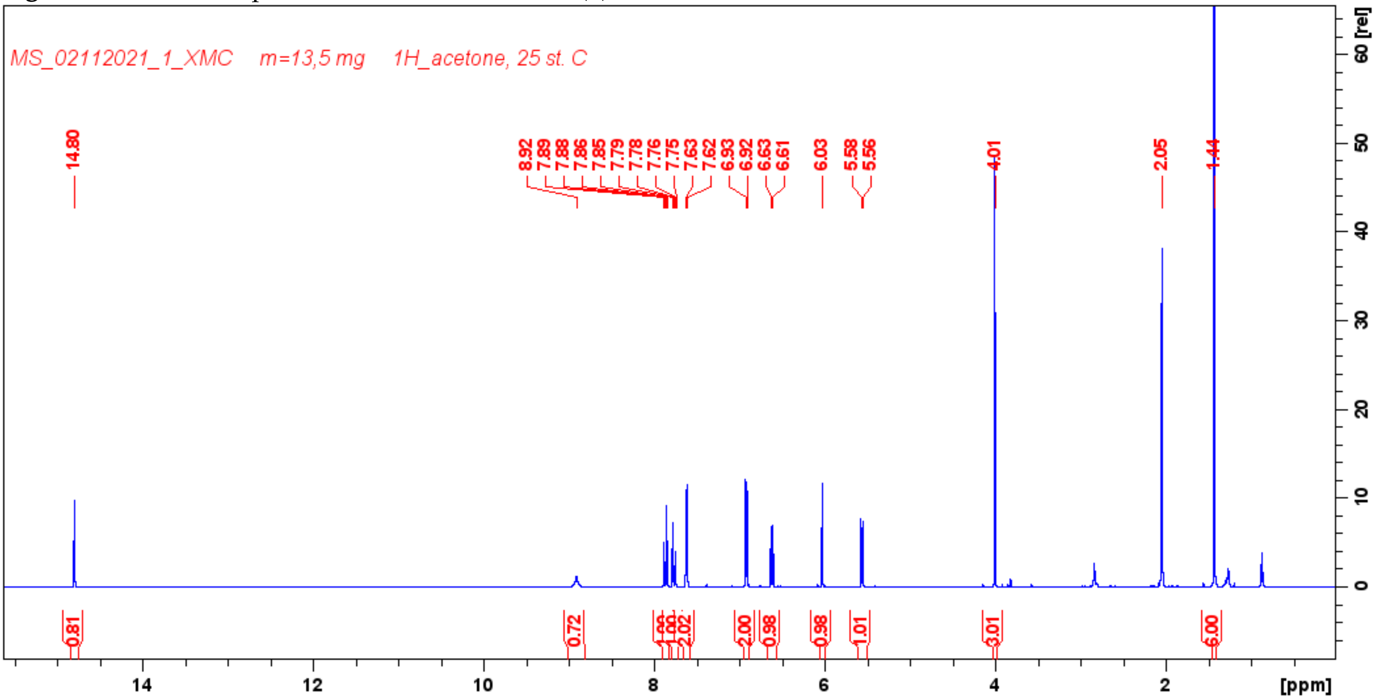

^{1}H NMR (500 MHz, acetone-d_6) δ: 14.80 (s, 1H, C2′-O*H*), 8.92 (brs, 1H, C4-O*H*), 7.88 (d, *J* 15.5, 1H, *H-α*), 7.77 (d, *J* 15.5, 1H, *H-β*), 7.62 (d, *J* 8.5, 2H, *H*-2, *H*-6), 6.92 (d, *J* 8.5, 2H, *H-3*, *H-5*), 6.62 (d, J 10.0, 1H, *H-1″*), 6.03 (s, 1H, H-5′), 5.57 (d, *J* 10.0, 1H, *H-2″*), 4.01 (s, 3H, C6′-OC*H*$_3$), 1.44 (s, 6H, *H-4″*, *H-5″*). Assignments in accordance with the literature data: Popłoński et.al. (31).

Figure S6. ^{13}C-NMR spectrum of xanthohumol C (**2**) in acetone-d_6:

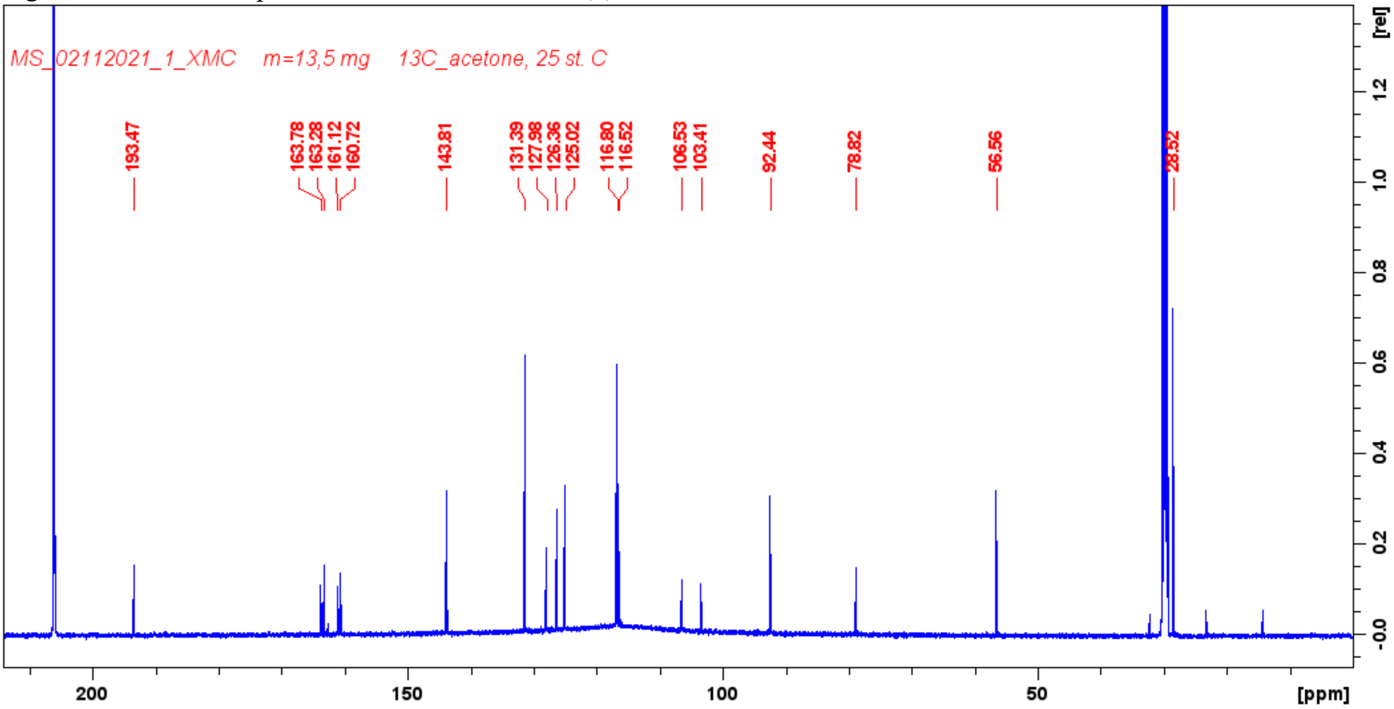

^{13}C NMR (125 MHz, acetone-d_6) δ:193.5 (*C*=O), 163.8 (*C-6′*), 163.3 (*C-2′*), 161.1 (*C-4′*), 160.7 (*C-4*), 143.8 (*H-β*), 131.4 (*C*-2, *C*-6), 128.0 (*C-1*), 126.4 (*C-2″*), 125.0 (*H-α*), 116.8 (*C-3*, *C-5*), 116.5 (*C-1*″), 106.5 (*C-1′*), 103.4 (*C-3′*), 92.4 (*C-5′*), 78.8 (*C-3*″), 56.6 (-OCH$_3$), 28.5 (*C-4*″, *C-5*″). Assignments in accordance with the literature data: Popłoński et.al. (31).

Figure S7. ^{1}H-NMR spectra of 1′′,2′′-dihydroxanthohumol C (**3**) in acetone-d_6:

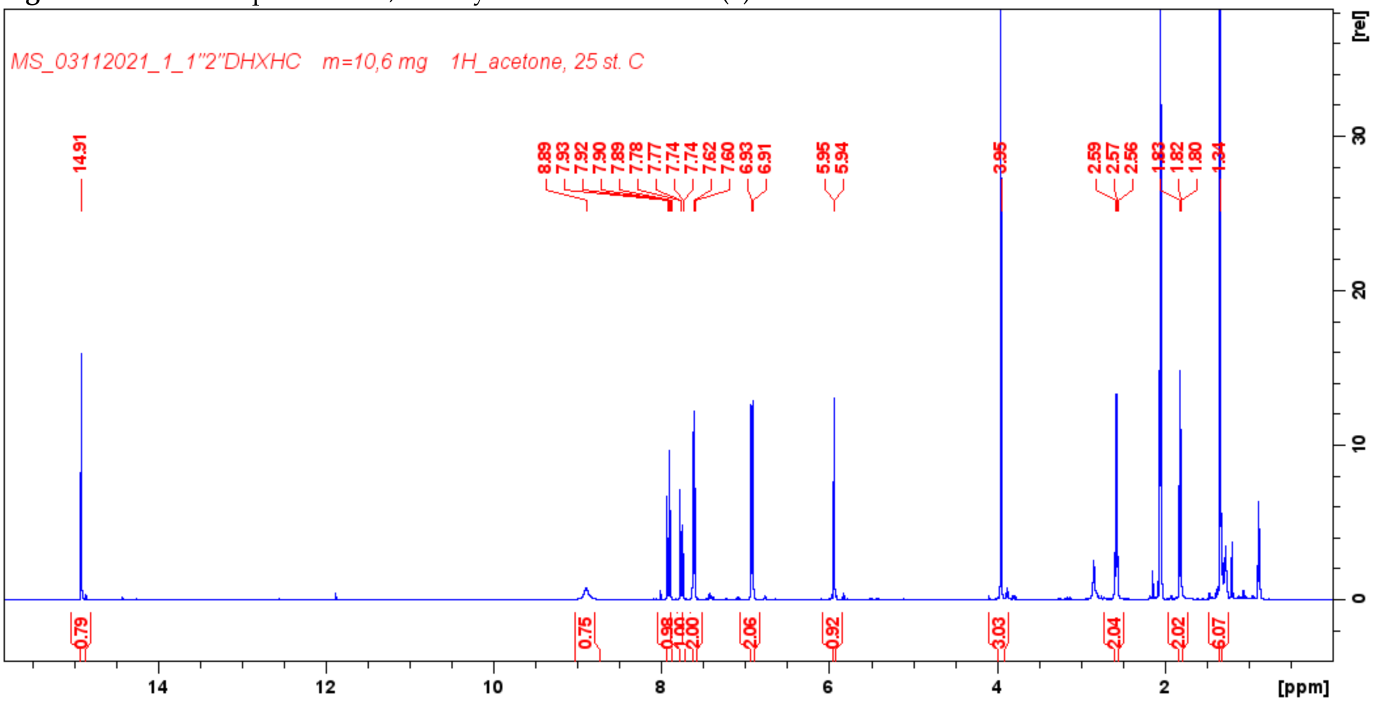

^{1}H NMR (500 MHz, acetone-d_6) δ: 14.91 (s, 1H, C2′-O*H*), 8.89 (s, 1H, C4-O*H*), 7.93 (d, *J* 15.5, 1H, *H-β*), 7.74 (d, *J* 15.5, 1H, *H-α*), 7.61, (d, J 8.5, 2H, *H-2*, *H-6*), 6.92 (d, *J* 8.5, 2H, *H-3*, *H-5*), 5.94 (s, 1H, *H-5′*), 3.85 (s, 3H, C6′-OCH_3), 2.57 (t, *J* 6.5, 2H, *H-1″*), 1.82 (t, *J* 6.5, 2H, *H-2″*), 1.34 (s, 6H, *H-4″*, *H-5″*). Assignments in accordance with the literature data: Popłoński et.al. (31).

Figure S8. ^{13}C-NMR spectra of 1′′,2′′-dihydroxanthohumol C (**3**) in acetone-d_6:

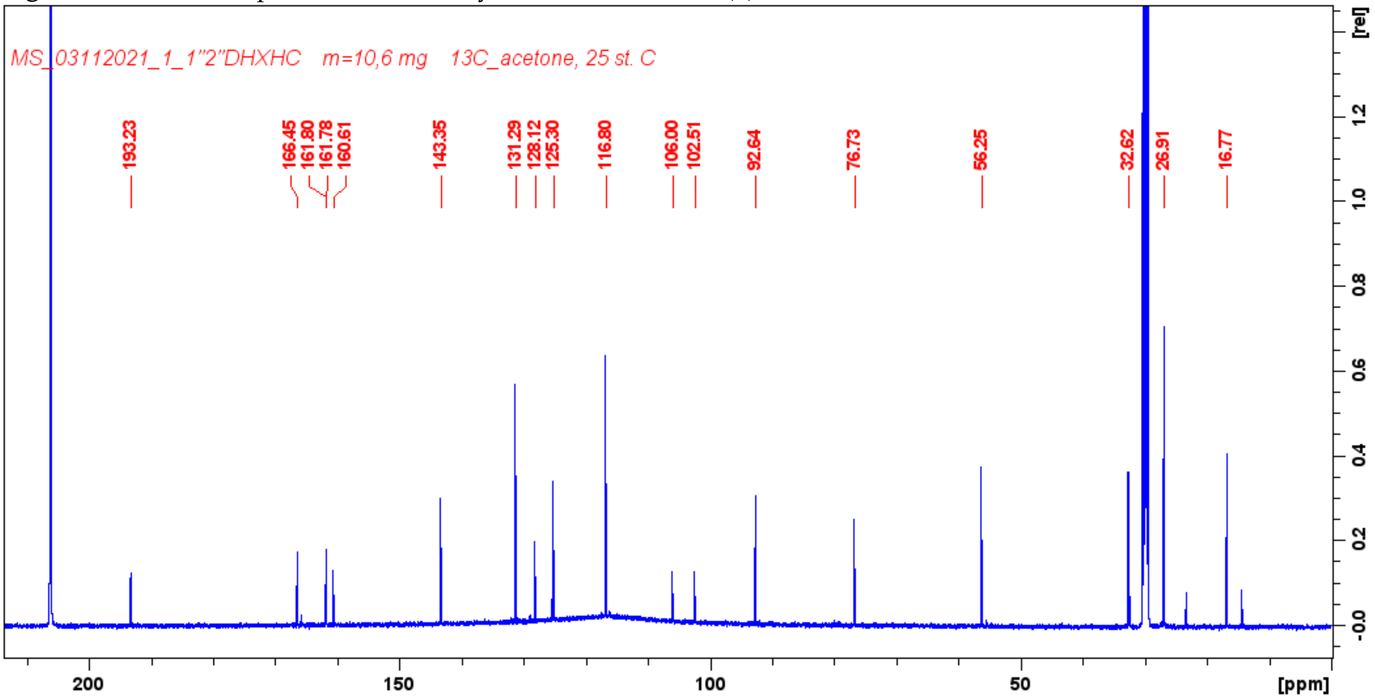

^{13}C NMR (125 MHz, acetone-d_6) δ: 193.2 (*C*=O), 166,4 (*C-4*), 161.8 (*C-2′, C-6′*), 160.6 (*C-4′*), 143.3 (*C-β*), 131.3 (*C-2, C-6*), 128.1 (C-1), 125.3 (*C-α*), 116.8 (*C-3, C-5*), 106.0 (*C-1′*), 102.5 (*C-3′*), 92.6 (*C-5′*), 76.7 (*C-3″*), 55.3 (-OCH_3), 32.6 (*C-2″*), 26.9 (*C-4″, C-5″*), 16,8 (*C-1″*). ″). Assignments in accordance with the literature data: Popłoński et.al. (31).
